# Microbial inhibitors of the fungus *Pseudogymnoascus destructans*, the causal agent of white-nose syndrome in bats

**DOI:** 10.1371/journal.pone.0179770

**Published:** 2017-06-20

**Authors:** Emma W. Micalizzi, Jonathan N. Mack, George P. White, Tyler J. Avis, Myron L. Smith

**Affiliations:** 1 Department of Biology, Carleton University, Ottawa, Ontario, Canada; 2 RIFDS Inc., Ottawa, Ontario, Canada; 3 Department of Chemistry, Carleton University, Ottawa, Ontario, Canada; Wadsworth Center, UNITED STATES

## Abstract

*Pseudogymnoascus destructans*, the fungus that causes white-nose syndrome in hibernating bats, has spread across eastern North America over the past decade and decimated bat populations. The saprotrophic growth of *P*. *destructans* may help to perpetuate the white-nose syndrome epidemic, and recent model predictions suggest that sufficiently reducing the environmental growth of *P*. *destructans* could help mitigate or prevent white-nose syndrome-associated bat colony collapse. In this study, we screened 301 microbes from diverse environmental samples for their ability to inhibit the growth of *P*. *destructans*. We identified 145 antagonistic isolates, 53 of which completely or nearly completely inhibited the growth of *P*. *destructans* in co-culture. Further analysis of our best antagonists indicated that these microbes have different modes of action and may have some specificity in inhibiting *P*. *destructans*. The results suggest that naturally-occurring microbes and/or their metabolites may be considered further as candidates to ameliorate bat colony collapse due to *P*. *destructans*.

## Introduction

*Pseudogymnoascus destructans* Minnis & D.L. Lindner is the fungus that causes a deadly disease in hibernating bats known as white-nose syndrome (WNS) [1]. *P*. *destructans* is believed to have been introduced to North America from Europe and was first discovered in New York in 2006 [2,3]. Over the past decade, *P*. *destructans* has caused extensive local extinctions and ten-fold reductions in affected North American bat populations [4]. Further, *P*. *destructans* has rapidly spread to 32 U.S. states and 5 Canadian provinces [3] and is predicted to continue spreading [5], potentially threatening over half of all North American bat species [3]. Such widespread loss of bats as prominent insectivores will undoubtedly have costly ecological, agricultural, and economic consequences [6].

*P*. *destructans* causes WNS by colonizing the skin of hibernating bats, creating lesions and increasing the frequency with which bats emerge from torpor. This is often lethal as the increased energy demands of disrupted torpor can result in dehydration and emaciation before water or food is available [7]. Further mortality may be caused by immune reconstitution inflammatory syndrome, where bats regain immune function after a period of hibernation-induced immunosuppression and have severe, lethal immune responses to *P*. *destructans* infection [8]. After the winter, surviving bats can rid themselves of *P*. *destructans* [9,10] and quickly heal their skin lesions [11]. However, because *P*. *destructans* persists in hibernacula by growing saprotrophically when bats are absent [12,13], it is possible that healthy bats could be infected when entering contaminated hibernacula [9,14]. While the role of the saprotrophic growth of *P*. *destructans* in the white-nose syndrome epidemic remains unclear, model predictions have suggested that under certain circumstances, reducing the growth of *P*. *destructans* in hibernacula may mitigate or prevent WNS-associated colony collapse [14,15]. Taken together, this suggests that targeting the growth of *P*. *destructans* in hibernacula may be an important part of managing the WNS epidemic.

In this study, we isolated a diversity of microbes from Ontario and Quebec, Canada, and screened for ones that inhibit *P*. *destructans* to identify potential biocontrol candidates and microbially-derived natural products that reduce the growth of *P*. *destructans* in a low temperature (hibernaculum-like) environment.

## Results

To screen for potential biocontrol candidates, we first obtained microbial isolates from local environmental samples and from culture collections. We co-inoculated one isolate per plate with *P*. *destructans* and classified each isolate based on an ability to inhibit *P*. *destructans* growth 14 days after inoculation (described in Methods).

**Fig 1:**
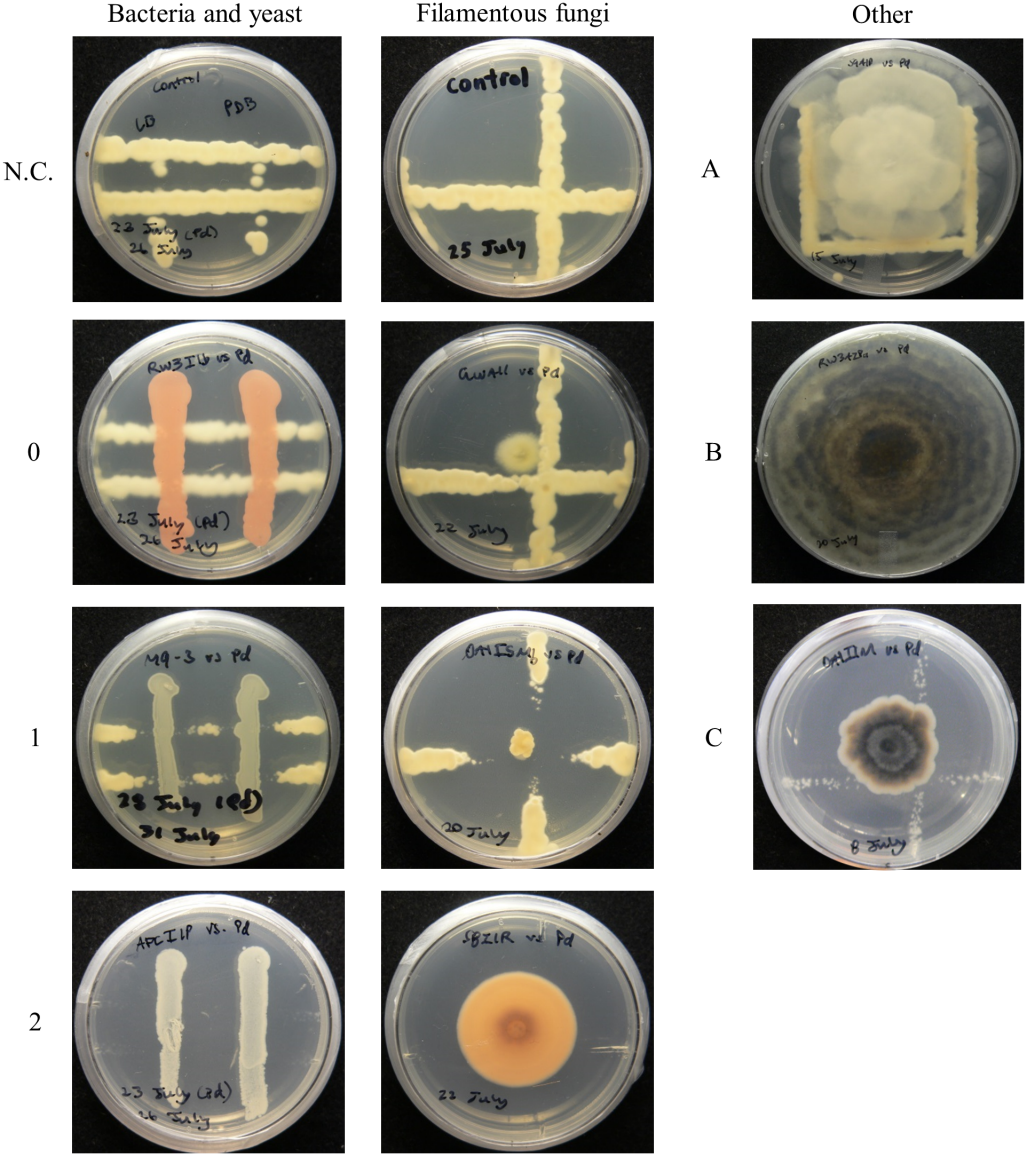
Representatives of each category of inhibition. *P*. *destructans* was inoculated on all plates as in the negative control (N.C.), and all photographs were taken 14 days after *P*. *destructans* inoculation. Classifications are (left and centre panels): (0) negligible (< 50%) inhibition, (1) considerable (50% to 85%) inhibition, (2) nearly complete/complete (> 85%) inhibition. Additionally, if applicable (right panel): (A) growth of the isolate is limited by *P*. *destructans*, (B) the isolate grew over *P*. *destructans* such that affected *P*. *destructans* colonies were no longer visible, (C) *P*. *destructans* colonies were present, but uniformly smaller than in the control plate.

Inhibition scores against *P*. *destructans* by each bacterial, filamentous fungal, and yeast isolate were calculated based on *P*. *destructans* colony area in the presence of isolates (see Methods) and classified as negligible (less than 50% inhibition of *P*. *destructans*), considerable (50% to 85% inhibition), or complete/nearly complete (greater than 85% inhibition), as summarized in Table 1 and represented in Fig 1. Nearly 50% of the 301 isolates examined were antagonistic to *P*. *destructans*, and over 15% completely or nearly completely inhibited growth of *P*. *destructans*. Most of the isolates that inhibited *P*. *destructans* did so by creating a zone of inhibition surrounding themselves where *P*. *destructans* did not grow. However, other modes of inhibition were also evident. For example, some fast-growing filamentous fungi grew over *P*. *destructans* colonies and induced a highly vacuolized appearance to *P*. *destructans* hyphae that was suggestive of programmed cell death (S1 Fig) [16], while the presence of some yeast isolates resulted in *P*. *destructans* colonies that remained uniformly small over the entire plate. For a tested subset of these inhibitory yeasts, this was a fungistatic effect since the inhibited *P*. *destructans* colonies resumed normal growth when transferred to a plate without the yeast. Only a few of the environmental isolates were inhibited by *P*. *destructans* (Table 1).

**Table 1:** Isolate inhibition of *P*. *destructans*.

	Bacteria	Filamentous fungi	Yeast	Total
Total screened	130	158	13	301
Negligible (< 50%) inhibition	75	75	6	156
Considerable (50% to 85%) inhibition	27	60	5	92
Complete/nearly complete (> 85%) inhibition	28	23	2	53
*P*. *destructans* inhibited antagonist	0	11	0	11
Antagonist grew over *P*. *destructans*	0	31	0	31
Reduced *P*. *destructans* colony size	8	25	5	38

Summary table showing the number of bacterial, filamentous fungal, and yeast isolates for each classification of inhibition 14 days after *P*. *destructans* inoculation.

Isolates that inhibited the growth of *P*. *destructans* are henceforth referred to as ‘antagonists’. The most inhibitory antagonists were taxonomically identified, where possible, to genus or species (see Methods, S1 Table). The 28 most effective bacterial antagonists were from the genera *Bacillus* (17 strains), *Pantoea* (3 strains), *Streptomyces* (3 strains), *Pseudomonas* (2 strains), and 1 strain each from *Rahnella*, *Arthrobacter*, and *Sphingobium*. The 23 most inhibitory filamentous fungi were primarily from the genera *Penicillium* (11 strains) and *Trichoderma* (7 strains), and the genera *Oidiodendron*, *Boeremia*, *Botrytis*, and *Phoma* each had 1 representative. One strongly inhibitory antagonistic filamentous fungus (isolate RW3A2Pa) could not be identified. Yeast belonging to *Cystofilobasidium* (2 strains) were also among the most inhibitory antagonists. All antagonists of *P*. *destructans* were preserved as frozen glycerol stocks at -80°C.

**Fig 2:**
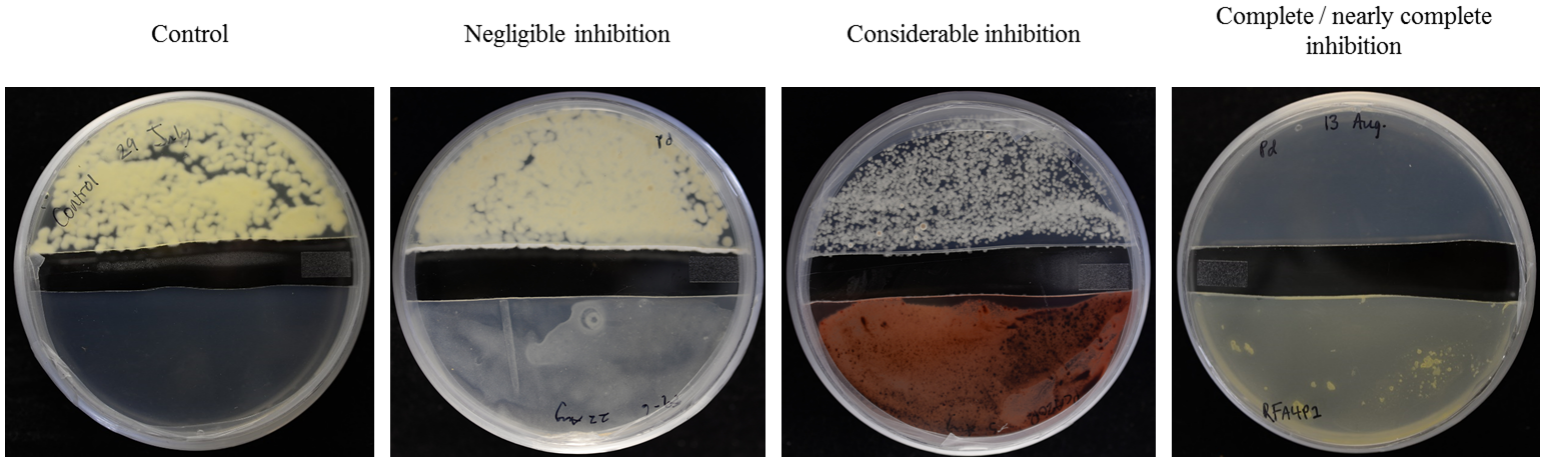
Evidence of inhibition of *P*. *destructans* by volatiles produced by antagonists. *P*. *destructans* and an antagonist were inoculated on separate pieces of agar within a single Petri plate. Photographs show *P*. *destructans* inoculated on the top and an antagonist on the bottom of the plate except for control plate (left), which contains *P*. *destructans* with no antagonist. Volatile production by antagonists was evident as considerable or complete growth inhibition of *P*. *destructans*. All photographs were taken 14 days after inoculation.

We examined if antagonists that completely or uniformly inhibited *P*. *destructans* acted through volatile compounds by inoculating *P*. *destructans* separately from antagonists in a shared airspace (see Methods). Based on these assays, 7 of 28 antagonists tested produced volatiles that effectively reduced the growth of *P*. *destructans* for 6 to 10 days after inoculation. Volatiles from 2 of these antagonists (*Oidiodendron* sp. PCA20P and *Pantoea* sp. OA1I3M) caused considerable (50–85%) inhibition and 2 (*Pantoea ananatis* RFA4P2 and *Cystofilobasidium capitatum* RW3I1a) caused complete or nearly complete (greater than 85%) inhibition of *P*. *destructans* at 14 days after inoculation (see S2 Table). This suggests that at low temperatures, these antagonists constitutively produce volatile compounds that inhibit *P*. *destructans*. A representative of each class of inhibition by volatiles is shown in Fig 2.

We conducted gas chromatography-mass spectrometry (GC-MS) to identify volatile compounds produced by the antagonists that inhibited *P*. *destructans* 14 days after inoculation. Volatile inhibitory compounds were identified in the headspace jars for three of the four antagonists that were analyzed with GC-MS. We detected 2-methyl-1-propanol for *C*. *capitatum* RW3I1a and *Pantoea* sp. OA1I3M. We also detected 2-methyl-1-butanol for *C*. *capitatum* RW3I1a. Propanoic acid and 1-pentanol were detected for *P*. *ananatis* RFA4P2. Other compounds appeared to be present in samples but could not be conclusively identified. *Oidiodendron* sp. PCA20P did not produce any detectable volatiles, suggesting that unidentified factors are required to induce volatile production in this antagonist. The most probable identification for each compound, along with the corresponding match scores and probability scores are included in S3 Table.

Compounds tentatively identified through GC-MS were screened in bioassays to determine their ability to inhibit *P*. *destructans*. All four compounds tested (2-methyl-1-propanol, 2-methyl-1-butanol, propanoic acid, and 1-pentanol) completely inhibited *P*. *destructans* growth for the 14-day experimental period when 100 μl of compound was added. Only propanoic acid was completely inhibitory when 10 μl of compound was applied. These compounds appear to be fungicidal since completely inhibited *P*. *destructans* did not resume growth after the compounds were removed. The ethanol control (carrier solvent for 10 μl 1-pentanol assay) also mildly affected *P*. *destructans* growth such that after 14 days, the average colony diameter was approximately 30% smaller than in the water control.

We next conducted bioassays to examine the inhibitory specificity of 36 of the most effective antagonists. For these, we repeated the bioassays that we performed with our original strain of *P*. *destructans* against two additional strains of *P*. *destructans* and representatives of the closely related species, *Pseudogymnoascus pannorum* and *Pseudogymnoascus roseus*. Of the 36 antagonists tested in bioassays, 35 inhibited both additional strains of *P*. *destructans*, with 29 causing complete or nearly complete inhibition in both strains. Only 16 antagonists inhibited growth of *P*. *roseus* and/or *P*. *pannorum*, with 3 of these causing complete or nearly complete inhibition in one or both strains (S4 Table).

We also qualitatively assessed whether our best antagonists could produce antimicrobial products that inhibit *P*. *destructans*. We grew 35 of our top antagonists in axenic liquid cultures and screened the spent media for activity against the above mentioned 5 *Pseudogymnoascus* strains and, in addition, *Saccharomyces cerevisiae*. Many of our antagonists produced inhibitory compounds and, similar to bioassays, *P*. *destructans* strains were considerably more sensitive than other species tested (S5 Table). Spent media from 18 of 35 antagonists tested inhibited at least one of the three *P*. *destructans* strains, and nine of these did not inhibit other *Pseudogymnoascus* species or brewer’s yeast.

Inhibition scores for the 27 antagonists that were tested in both bioassays and spent media screens were not always congruent and some antagonists appear to have context-dependent induction and/or effects of inhibitory compounds. For example, there were 13 antagonists that inhibited all three strains of *P*. *destructans* in bioassays, while the respective spent culture medium caused no inhibition of *P*. *destructans*. Conversely, there were seven antagonists that caused negligible inhibition of *P*. *roseus* and/or *P*. *pannorum* in bioassays, while their spent culture medium was inhibitory to *P*. *roseus* and/or *P*. *pannorum*. Nevertheless, it is notable that the culture medium from several antagonists, including *Phoma* sp. OA1I1M, *Sphingobium* sp. S8A4Cs, *Trichoderma harzianum* RW1A2P, and *Paecilomyces inflatus* PCA5P, caused very strong inhibition in all three strains of *P*. *destructans* while causing low inhibition of *P*. *roseus*, *P*. *pannorum*, and yeast. The inhibitory compounds produced constitutively by these antagonists could be considered further as candidate natural products to inhibit growth of *P*. *destructans* in bat hibernation sites.

## Discussion

The white-nose syndrome that is caused by *P*. *destructans* has decimated eastern North American bat populations and is spreading across the continent [1,3,5]. Saprotrophic growth of *P*. *destructans* in cool hibernacula may contribute to perpetuating the WNS epidemic [14] and thus may facilitate the infection or reinfection of healthy bats. In an effort to find biocontrol agents that will reduce the abundance of *P*. *destructans* in bat hibernacula, we isolated microbes from diverse environmental samples and tested them for inhibitory activity against *P*. *destructans*. We identified 145 microbes that inhibited the growth of *P*. *destructans* to some extent, and 53 of these completely or nearly completely inhibited *P*. *destructans*.

In the past decade since *P*. *destructans* was discovered, only a few microbes capable of inhibiting *P*. *destructans* have been identified: *Rhodococcus rhodochrous* [17], *Trichoderma polysporum*, *Trichoderma harzianum* [18], and *Pseudomonas* spp. [19]. These microbes are being studied for their biocontrol potential, and our study adds nearly 150 candidate biocontrol agents to this list, Additionally, we expand the list of natural products [18,20–23] that could be of potential use in controlling *P*. *destructans* in bat caves. We also report on 4 volatile organic compounds that appear to have a fungicidal effect on *P*. *destructans*. All 4 of these compounds have previously been reported to be produced by fungi [e.g. 24–26] and to have at least some degree of inhibitory activity against fungi [e.g. 27–30]. Although differences in methodologies preclude making quantitative comparisons between the antagonists identified in this study and the antagonists of *P*. *destructans* that have been previously identified, the most notable finding from our study is the relatively high frequency of microbes that we identified that cause complete or nearly complete inhibition of *P*. *destructans*. While few of the previously identified antagonists have completely inhibited growth of P. destructans, we identified five bacterial and four filamentous fungal isolates that did so in bioassays or volatile tests. Additionally, while a bacterium has been reported that inhibits *P*. *destructans* through volatiles [17], we novelly identify microbes that produce inhibitory volatiles without induction.

The microbes that we identified have several promising features as biocontrol candidates in eastern North America. First, most of our top antagonists are microbes that already occur in Ontario and Quebec that present a lower risk than introducing foreign, potentially invasive, species. Second, many of our top antagonists displayed antifungal activity at about 13°C—a temperature that represents the average temperature of North American bat hibernacula. Third, several of our top antagonists constitutively secrete compounds that inhibit *P*. *destructans* at concentrations that are non-inhibitory to close relatives and to *S*. *cerevisiae*, suggesting the possibility that these antifungal agents may have some degree of specificity towards *P*. *destructans*. In addition, in both bioassays and liquid media screens, *P*. *destructans* was more sensitive to inhibition than close relatives, again suggesting the possibility that there may be a reduced risk of non-target effects from our antagonists. Fourth, we identified microbes that inhibit the growth of *P*. *destructans* by seemingly different modes. For example, *Trichoderma* and fast-growing filamentous fungal antagonists typically grew over and appeared to induce programmed cell death of *P*. *destructans* [16], indicating cell proximity/contact as a main mode of action, whereas most of our top antagonists secreted water-soluble inhibitors, indicating antibiosis as a main mode of action. Several antagonists also produced volatiles at hibernaculum-like temperatures that inhibit *P*. *destructans*. A diversity of modes of action provides the possibility of creating a stable biocontrol strategy that targets *P*. *destructans* through multiple mechanisms. Finally, our high success rate of isolating native biocontrol candidates suggests that as *P*. *destructans* continues to spread across the continent, additional local biocontrol candidates can be identified that may reduce the growth and persistence of *P*. *destructans* in hibernacula.

Another interesting aspect of our findings is that several antagonists that we identified are not known to produce antifungal compounds. To our knowledge, antifungals have not been characterized from species of *Boeremia*, *Phialosimplex*, *Ramularia*, or *Sphingobium*, all of which secreted inhibitors of *P*. *destructans*. Similarly, only preliminary characterizations of antifungals are reported for species of *Oidiodendron* [31] and *Cystofilobasidium* [32]. This suggests the possibility that some of our top antagonists may produce novel antifungals, which could have applications both within and beyond controlling *P*. *destructans*.

Although we identified many potential biocontrol agents of *P*. *destructans*, an important limitation of this study is that it only addressed inhibition of *P*. *destructans* under controlled laboratory conditions. Future challenges to developing a biocontrol of white nose syndrome are to find antagonistic organisms that selectively inhibit growth of *P*. *destructans* in natural hibernacula. Considering the high proportion of microbes that inhibited *P*. *destructans* in our tests, it is surprising that a biocontrol of *P*. *destructans* has not arisen naturally. It is possible that the effects of natural antagonists are limited by an insufficient abundance and nutritional augmentation of hibernaculum sediment may be necessary to support greater antagonist growth. To explore this further, we are now examining *P*. *destructans*-antagonist interactions in hibernaculum-like soil microcosms. Future research will also assess the synergistic effects of multiple antagonistic organisms towards *P*. *destructans*.

## Conclusions

We identified over 100 microbes that inhibit the growth of *P*. *destructans* in a low-temperature laboratory setting. These antagonistic microbes inhibit *P*. *destructans* with secreted compounds, by contact inhibition, or through volatiles. Future research is needed to validate potential biocontrol strategies under hibernaculum conditions. Our results suggest that local microbes can be a source of candidate biocontrol agents to reduce the abundance of the causal agent of white-nose syndrome in bat hibernation sites and remediate bat colony collapse.

## Materials and methods

### *Pseudogymnoascus* strains

*P*. *destructans* strains US-15, SH-864, and SH-991 were obtained from Agriculture and Agrifood Culture Collection, Ottawa, ON, Canada. Unless specified otherwise, all assays used *P*. *destructans* strain US-15. *P*. *roseus* S8A2CN and *P*. *pannorum* S8A5ACS1 were isolated from soil samples in Gatineau, Québec. All cultures were grown at 13 ± 1°C, within the optimal temperature range for *P*. *destructans* [33]. *Pseudogymnoascus* cultures were stored in Potato Dextrose Broth (PDB) amended with 15% sterile glycerol at -80°C.

### Isolation of antagonists

Antagonists used in this study were from various sources and locations as listed in S1 Table. Axenic antagonist cultures were grown in 5 ml Potato Dextrose Agar (PDA) slants and stored in PDB amended with 15% sterile glycerol at -80°C. Soil samples were collected from cold soils (0–10°C) in April 2015 from Ottawa, Gatineau, and Toronto areas and frozen at -20°C until use. Approximately 1.5 g of soil was mixed with 10 ml of sterile tap water and 10- to 1000-fold dilutions were plated onto various media including plates containing 0.2% chitin and 0.1× Vogel’s salts [34] to isolate chitinolytic fungi [35,36], LB Miller agar with 150 mg L^-1^ cycloheximide to isolate bacteria and select against fungi [37], PDA with 70 mg L^-1^ Rose Bengal to isolate slow-growing fungi [38], PDA with 100 mg L^-1^ ampicillin, 50 mg L^-1^ chloroamphenicol, and 75 mg L^-1^ streptomycin sulphate to isolate fungi without bacteria, and PDA alone to culture microbes non-specifically. Antagonists were also isolated from decomposing wood, birch bark, and hay. Additional antagonists were obtained from foam taken from the surface of the Rideau River (Ottawa, ON) and by leaving Petri plates open to the air. Further antagonists were isolated from compost and compost tea [39,40] and from environmental samples isolated with DG-18 (Dichloran-Glycerol Agar) in the Ottawa region. Several strains of *Bacillus* and *Trichoderma* were from the culture collection of M.L. Smith. [41,42].

### Bioassay for filamentous fungal and actinobacterial antagonists

Aliquots of *P*. *destructans* cultures, macerated with a Waring blender and stored at -80°C in PDB with 15% glycerol, were thawed at room temperature and diluted in PDB to 5000 CFU/ml in a sterile Eppendorf multichannel pipette reagent trough. A 50 × 75-mm flame-sterilized glass microscope slide was dipped edgewise into the trough and used to stamp a narrow line of *P*. *destructans* inoculum onto the surface of PDA in a Petri plate. Perpendicular lines were stamped on PDA at an appropriate distance from the centre in a 55-mm (for assaying slow growing antagonists) or 90-mm (for fast growing antagonists) Petri plate so that a given antagonist, inoculated in the centre of the plate, would contact *P*. *destructans* at about day seven. Control plates were created by stamping *P*. *destructans* inoculum without an antagonist.

### Bioassay for bacterial and yeast antagonists

PDA in 55-mm diameter Petri plates was pre-inoculated with *P*. *destructans* using a glass slide as described above to create two parallel lines of inoculum. Three days after *P*. *destructans* was inoculated, 7-μl aliquots of a log phase antagonist culture in LB (bacteria) or PDB (yeast) were streaked in duplicate through both lines of *P*. *destructans*. LB broth and PDB were streaked through *P*. *destructans* on replicate plates as controls.

### Assessing inhibition

Assay plates from antagonist screenings were photographed 14 days after *P*. *destructans* inoculation. Inhibition was quantified using image analysis to calculate the area of *P*. *destructans*. Images were scaled based on the size of the Petri plates and antagonist diameter was measured using the measure feature in ImageJ [43]. To aid in distinguishing *P*. *destructans* from background features, images were cropped to exclude as much background as possible and only one line of *P*. *destructans* was considered. Ilastik version 1.2.0 [44] was used to distinguish *P*. *destructans* colonies from antagonist colonies and background. Simple segmentations for each image were exported to ImageJ where the greyscale image was thresholded with a value of 2 for each parameter. *P*. *destructans* colony area was calculated using the analyze particles feature of ImageJ with no parameters specified for size or circularity. Thresholded images were manually checked to ensure *P*. *destructans* was fully and exclusively detected and the reported area for *P*. *destructans* was the area of one streak multiplied by 2 or 3, for plates stamped in duplicate or triplicate, respectively. Inhibition scores for each antagonist were reported as percent inhibition of *P*. *destructans*, relative to a no-antagonist control. Percent inhibition was calculated as $\left(1-\frac{Area_{treatment}}{Area_{control}}\right)\times \;100,$ where Area_treatment_ refers to the area of *P*. *destructans* in the presence of the antagonist and Area_control_ refers to the area of *P*. *destructans* in the no-antagonist control. The percent inhibition for each antagonist, along with the respective day 14 area of *P*. *destructans* and antagonist diameter are provided in S1 Table. The inhibition scores of *P*. *destructans* by an antagonist were ranked as 0 = negligible or no (less than 50%) inhibition of *P*. *destructans*; 1 = considerable (between 50% and 85%) inhibition of *P*. *destructans*; or 2 = complete or nearly complete (greater than 85%) inhibition of *P*. *destructans*. Additionally, if applicable, antagonistic ranks were qualified with: A = *P*. *destructans* inhibited the antagonist; B = the antagonist grew over *P*. *destructans* such that *P*. *destructans* colonies were no longer visible; and/or C = *P*. *destructans* colonies were present, but considerably smaller than colonies on the control plates. A representative of each of these classifications and a control plate is shown in Fig 1.

### Identification

Isolates that inhibited *P*. *destructans* were identified, including all isolates that caused greater than 85% inhibition, by sequences of *ITS* rDNA (fungi), *16S* (bacteria) rDNA, and *beta-tubulin* (*Penicillium* sp.) DNA. Morphological identification was used to augment sequence-based identifications of filamentous fungi.

#### DNA extraction

DNA was extracted using a modified form of the protocol outlined by Lõoke and colleagues [45]. Antagonists were grown at room temperature in a 1.5-ml epitube containing 1 ml sterile PDB until the culture was visible throughout the tube. The cells were pelleted by centrifugation and resuspended in 100–150 μl of 200 mM lithium acetate with 1% SDS. Approximately 15 mg of 0.5 mm (for filamentous fungi or yeast) or 0.1 mm (for bacteria) glass beads were added to each epitube before the tubes were placed into a Fisher Scientific Isotemp waterbath at 70°C for 10 minutes and subsequently cooled on ice. The epitubes were then shaken in a Retsch MM301 mixer mill at 20 Hz twice for 2 minutes each, separated by a 2-minute pause, and then 300 μl of ice-cold 95% ethanol was added before each tube was vortexed and left for 10 minutes. The epitubes were then centrifuged at 15,000 rpm for 3 minutes and the pellet was rinsed with 70% ethanol and dried in a Savant Speed Vac Concentrator before resuspending in 100 μl of distilled water. The epitubes were centrifuged at 15,000 rpm for approximately 15 seconds and 20 μl of the supernatant was removed and stored at -20°C until use in PCR amplifications.

#### PCR and DNA sequencing

The *ITS* region of filamentous fungi and yeast was amplified using ITS5 (5’-GGAAGTAAAAGTCGTAACAAGG-3’) or ITS9mun (5’-TGTACACACCGCCCGTCG-3’) forward primers and ITS4 (5’-TCCTCCGCTTATTGATATGC-3’) reverse primer [46–48]. The *16S* region of bacterial samples was amplified using Bakt_341F (5’-CCTACGGGNGGCWGCAG-3’) forward and Bakt_805R (5’-GACTACHVGGGTATCTAATCC-3’) reverse primer [49,50]. The *beta-tubulin* gene of *Penicillium* antagonists was amplified using Bt2a (5’-GGTAACCAAATCGGTGCTGCTTTC-3’) forward primer and Bt2b (5’-ACCCTCAGTGTAGTGACCCTTGGC-3’) reverse primer [51]. Standard PCR reactions contained approximately 2 μl each of 10 μM forward and reverse primers, 5 μl of 10× Taq buffer (BioShop, Burlington, ON), 2 μl of 25 mM MgCl_2_, 2 μl of 10 mM dNTPs (New England BioLabs, Whitby, ON), 1.25 units of Taq (New England BioLabs), approximately 100 ng of template DNA, and sterile Milli-Q water to 50 μl. For filamentous fungal and yeast *ITS*, the PCR schedule was 5 minutes at 94°C, then 35 cycles each with 30 seconds at 94°C, 56°C, and 72°C, then 7 minutes at 72°C. For bacterial *16S*, the schedule was 10 minutes at 94°C, then 35 cycles each with 60 seconds at 94°C, 57°C, and 72°C, then 10 minutes at 72°C. The *beta-tubulin* PCR schedule was 3 minutes at 95°C, then 35 cycles each with 30 seconds at 95°C, 60°C, and 72°C, then 7 minutes at 72°C. PCR products were purified using a Geneaid PCR DNA fragments extraction kit and sent to Génome Québec (Montréal, QC) for Sanger sequencing (Applied Biosystems—3730xl DNA Analyzer). Forward and reverse sequences were aligned using ExPASy ClustalW and the NCBI nucleotide BLAST database was used to identify the microbes. DNA sequences were submitted to GenBank and accession numbers are given in S1 Table.

### Assessing volatile production

#### Shared airspace experiments

A shared-airspace experiment was performed to assess if any of the antagonists that completely or uniformly inhibited *P*. *destructans* in bioassays acted through volatiles. A strip approximately 1 cm wide was cut out of the centre of a 90-mm PDA plate to create two separated pieces of agar. A thawed stock of *P*. *destructans* was diluted in PDB and approximately 1.3 × 10^4^ CFUs were spread onto agar on one side of the plate. A small amount of antagonist (a small loopful for bacteria/yeast or a needleful for filamentous fungi) was suspended in 250 μl of liquid medium (PDB for filamentous fungi and yeast, LB for bacteria) and 200 μl of this suspension was spread onto the agar surface opposite of *P*. *destructans*. The plates were sealed with Parafilm and incubated at 13 ± 1°C. The growth of *P*. *destructans* was monitored and compared to a control without an antagonist from 6 to 14 days after inoculation. The day 14 area of *P*. *destructans* and respective inhibition scores for each antagonist were calculated using ilastik [44] and ImageJ [43], as above. Based on inhibition scores, antagonist inhibition of *P*. *destructans* was scored as negligible, considerable, or complete/nearly complete, as described above.

#### Volatile identification

Headspace gas chromatography mass spectrometry (GC-MS) was used to analyze volatiles produced by the four microbes that had contact-independent inhibition of *P*. *destructans* after 14 days. *Pantoea ananatis* RFA4P2, *Oidiodendron* sp. PCA20P, *Pantoea* sp. OA1I3M, *Cystofilobasidium capitatum* RW3I1a, and a no-antagonist (blank) control were inoculated on 3-ml PDA slants inside headspace jars and grown for 5 days at 13 ± 1°C. The headspace jars were covered with a double layer of sterile foil for the incubation period and sealed approximately 10 minutes before performing GC-MS. The GC-MS was done with an Agilent Technologies 7697A headspace sampler coupled to an Agilent Technologies 7820A gas chromatography system and an Agilent Technologies 5977E mass spectrometer detector. The vials were sampled at 33.9°C, the loop temperature was 45°C and the transfer line was 80°C. Samples were injected for gas chromatography in splitless mode. A 30 m × 250 μm × 0.5 μm DB-WAXetr column was used with helium carrier gas at a constant flow rate of 1 ml/min. The oven temperature was held at 50°C for 2 min and then increased at a rate of 10°C/min to 235°C, where it was held for 5.5 minutes. Mass-spectrometry was performed with electron ionization, and identification of volatile compounds was performed by comparison to version 5 of the National Institute of Standards and Technology (NIST) spectra database.

Compounds identified by GC-MS were tested for inhibitory activity against *P*. *destructans* in bioassays. Approximately 2.6 × 10^4^ CFUs of *P*. *destructans* were inoculated onto the surface of 15 ml of PDA in a 90-mm diameter Petri dish. Petri dishes were inverted and a 2.5-cm diameter sterile Whatman 3 paper disc was placed on the lid of each dish. The filter paper was saturated with 10 or 100 μl of 2-methyl-1-propanol (J.T. Baker Chemical Co., Phillipsburg NJ), 2-methyl-1-butanol (Sigma-Aldrich, Oakville ON), propanoic acid, or 1-pentanol (BDH Chemicals, Toronto ON). The 10 μl aliquots were diluted to 100 μl in sterile distilled water, except for 1-pentanol, which was diluted in 95% ethanol. Separate assays were done using 100 μl/disc of water or ethanol as carrier controls. Plates were sealed with Parafilm and incubated inverted at 13 ± 1°C for 14 days and growth of *P*. *destructans* was assessed. To test whether the inhibition of *P*. *destructans* was fungistatic or fungicidal, the paper discs were removed on day 14 and the lid was dried with a sterile Kimwipe. Plates were sealed with Parafilm and incubated at 13 ± 1°C for an additional 14 days, after which growth of *P*. *destructans* was assessed.

### Assessing activity of spent antagonist media

We screened antagonists for production of antimicrobial compounds that inhibit *P*. *destructans*. We used spent culture media from a subset of antagonists of *P*. *destructans* US-15 and screened these against *P*. *destructans* strains US-15, SH-864, and SH-991, *P*. *roseus* S8A2CN, *P*. *pannorum* S8A5ACS1, and *Saccharomyces cerevisiae* strain S288C. Thirty-five antagonists were each grown stationary in 250 ml flasks with 50 ml of PDB (fungi) or LB (bacteria) for 4 weeks at 13 ± 1°C. After this time, the culture medium was harvested and passed through a 0.2-μm syringe filter and then 10× concentrated following lyophilisation. To assess inhibition of *Pseudogymnoascus* spp. and yeast, 50 μl of cell-free filtrate was 1:1 serially diluted in 50 μl of PDB (for *Pseudogymnoascus* spp.) or YPD (10 g L^-1^ yeast extract, 20 g L^-1^ peptone, 20 g L^-1^ D-glucose; for yeast) in a 96-well microtiter plate before the addition of 150 μl of PDB with approximately 100 CFUs *Pseudogymnoascus* sp. or YPD with approximately 150 yeast cells. Ten-times concentrated medium (PDB or YPD, as appropriate) was used as a carrier control for antagonist filtrates. Inhibition was assessed visually after 14 days of growth at 13 ± 1°C (*Pseudogymnoascus* sp.) or 3 days of growth at 30°C (*S*. *cerevisieae*) and was defined as the lowest concentration of spent medium at which no growth was visible.

### Tests for specificity of inhibitory interactions

To test the specificity of the inhibition of *P*. *destructans*, we repeated bioassays using 36 of our top antagonists against two additional strains of *P*. *destructans* (SH-864 and SH-991) and two close relatives of *P*. *destructans* (*P*. *roseus* S8A2CN and *P*. *pannorum* S8A5ACS1). The bioassays were conducted as described above for *P*. *destructans* US-15, however the concentration of *Pseudogymnoascus* spp. inoculum stamped onto the plates was adjusted for each strain so that after 14 days a continuous line of fungal mycelium was visible for each strain. The concentrations were approximately 11,300 CFU/ml *P*. *destructans* SH-864, 10,000 CFU/ml *P*. *destructans* SH-991, 11,100 CFU/ml *P*. *roseus* S8A2CN, and 8,700 CFU/ml *P*. *pannorum* S8A5ACS1. Inhibition scores were calculated and ranked as described for *P*. *destructans* bioassays.

## Supporting information

S1 Fig Representative image showing *P*. *destructans* vacuolization near antagonist hyphae. *P*. *destructans* was pre-inoculated for one week on a PDA-coated microscope slide before the slide was also inoculated with *Penicillium crustosum* BWA2P. After 4 days of antagonist growth, *P*. *destructans* hyphae had a healthy appearance on the colony side away from the antagonist (A), but had a vacuolized appearance suggestive of programmed cell death near antagonist hyphae (B). Scale bar represents 100 μm.
(TIF)

S1 Table Identification of microbial antagonists of *Pseudogymnoascus destructans*. Isolates are sorted by percent inhibition, which reflects the degree of inhibition of *P*. *destructans* by an antagonist. Isolates were ranked as follows: 0 = negligible (< 50%) inhibition, 1 = considerable (50% to 85%) inhibition, 2 = complete or nearly complete (> 85%) inhibition. Additionally, if applicable, ranks were qualified with: A = growth of the antagonist is limited by *P*. *destructans*, B = the antagonist grew over *P*. *destructans* such that affected *P*. *destructans* colonies were no longer visible, C = *P*. *destructans* colonies were present, but uniformly smaller than in the control plate. *P*. *destructans* area and isolate diameter refer to sizes 14 days after *P*. *destructans* was inoculated. Genbank accession numbers refer to sequences of 16S rDNA (bacteria), ITS rDNA (fungi), and beta-tubulin DNA (*Penicillium* sp.).
(XLSX)

S2 Table Screen for inhibition of *Pseudogymnoascus destructans* by volatiles produced by selected antagonists. *P*. *destructans* and an antagonist were inoculated on separate pieces of agar within a single plate and incubated at 13 ± 1°C. Inhibition was assessed at 6, 10 and 14 days after inoculation and percent inhibition was calculated on day 14 as (1 − Area_treatment_/Area_control_) × 100. The average *P*. *destructans* area in no-antagonist controls was 2043.85 mm^2^. Antagonists were ranked as 0 = negligible (< 50%) inhibition, 1 = considerable (50% to 85%) inhibition, or 2 = complete or nearly complete (> 85%) inhibition, based on the area of *P*. *destructans* in the presence of the antagonist (treatment) compared to in the no-antagonist control. Asterisks (*) indicate cases where more pronounced inhibition of *P*. *destructans* was evident at day 6 and 10 but decreased by day 14.
(XLSX)

S3 Table Most probable identifications for each volatile compound detected through gas chromatography-mass spectrometry analysis of fungal and bacterial antagonists. Antagonists were inoculated on 3-ml PDA slants inside of headspace jars and volatiles were identified. Also listed is the corresponding retention time, molecular formula, match factor (MF), reverse match factor (RMF), probability of match (Prob), and in library (InLib) score for each proposed identification. Italicized entries were tested against *P*. *destructans* in further assays.
(XLSX)

S4 Table Inhibition scores for 36 selected antagonists against different *Pseudogymnoascus* species. Two strains of *P*. *destructans* and one strain each of *P*. *roseus* and *P*. *pannorum* were used. Percent inhibition was calculated as (1 − Area_control_ / Area_experimental_) × 100. *Pseudogymnoascus* spp. areas refer to the total area of *Pseudogymnoascus* spp. 14 days after inoculation. Antagonists were ranked as: 0 = negligible (< 50%) inhibition, 1 = considerable (50%–85%) inhibition, 2 = nearly complete/complete (> 85%) inhibition, relative to a no-antagonist control. Additionally, if applicable, ranks were qualified with: A = growth of the antagonist is limited by *Pseudogymnoascus* spp., B = the antagonist grew over colonies such that affected *Pseudogymnoascus* colonies were no longer visible, C = *Pseudogymnoascus* colonies were present, but uniformly smaller than in the control plate.
(XLSX)

S5 Table Inhibitory concentrations for the filtered, spent media from each of 35 antagonist cultures. Antagonists were grown at 13 ± 1°C for 28 days and screened against three strains of *Pseudogymnoascus destructans* and one strain each of *P*. *roseus*, *P*. *pannorum*, and *Saccharomyces cerevisiae*. Inhibitory concentrations were recorded after 14 days of *Pseudogymnoascus* growth or 3 days of *S*. *cerevisiae* growth and are expressed relative to the original concentration of the day 28 antagonist medium. NI indicates that complete inhibition was not evident even at highest concentration of 2.5× antagonist medium and ND indicates that inhibitory concentration was not assessed.
(XLSX)
