# Supplementary figures and images for: Microbial inhibitors of the fungus Pseudogymnoascus destructans, the causal agent of white-nose syndrome in bats
Source: PLoS One. 2017 Jun 20;12(6):e0179770. doi: 10.1371/journal.pone.0179770 (PMC5478148; doi:10.1371/journal.pone.0179770)

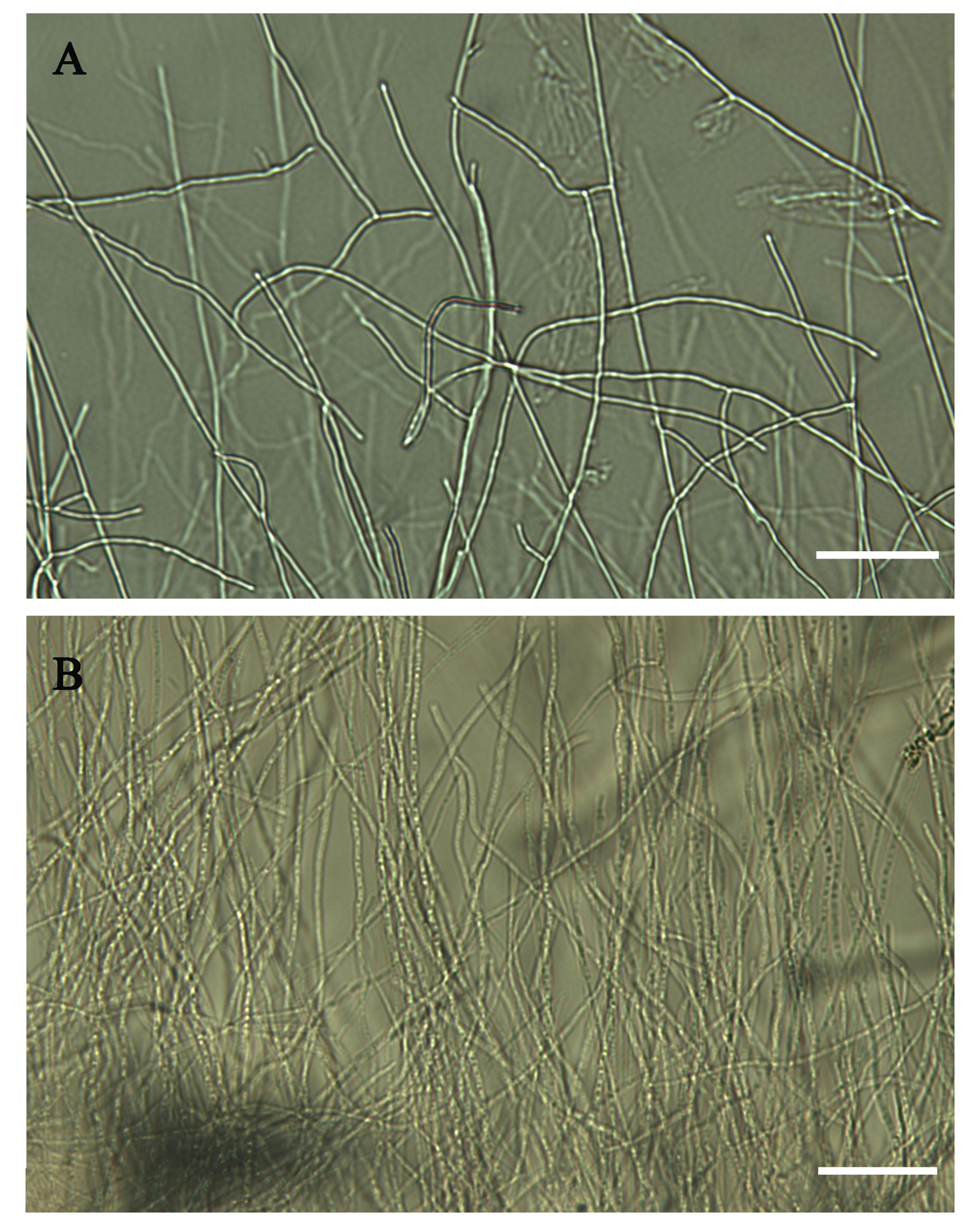

Supplement: S1 Fig — P. destructans was pre-inoculated for one week on a PDA-coated microscope slide before the slide was also inoculated with Penicillium crustosum BWA2P. After 4 days of antagonist growth, P. destructans hyphae had a healthy appearance on the colony side away from the antagonist (A), but had a vacuolized appearance suggestive of programmed cell death near antagonist hyphae (B). Scale bar represents 100 μm. (TIF) [file pone.0179770.s001.tif]
